# Achalasia as a complication of bulimia nervosa: A case report

**DOI:** 10.4102/sajpsychiatry.v23.996

**Published:** 2017-02-07

**Authors:** Meryem O. Kutuk, Gulen Guler, Ali E. Tufan, Fevziye Toros, Umut Kaytanli

**Affiliations:** 1 Department of Child and Adolescent Psychiatry, Baskent University, Turkey; 2 Department of Child and Adolescent Psychiatry, Elazig Mental Health Hospital, Turkey; 3 Department of Child and Adolescent Psychiatry, Abant İzzet Baysal University, Turkey; 4 Department of Child and Adolescent Psychiatry, Mersin University, Turkey; 5 Department of Child and Adolescent Psychiatry, Zeynep Kamil Women and Children’s Diseases Education and Research Hospital, Turkey

## Abstract

**Objective:**

Oesophageal achalasia is a medical condition characterised by oesophageal aperistalsis, an increased resting pressure with partial or incomplete relaxation of the lower oesophageal sphincter. Bulimia nervosa (BN) is an eating disorder manifested by binge eating attacks followed by recurrent inappropriate compensatory behaviours, such as self-induced vomiting and excessive exercise. Dysphagia, regurgitation, vomiting, retrosternal pain, heartburn, weight loss, avoidance of eating, consumption of large amount of liquids and aberrant eating behaviours are symptoms of both achalasia and BN. Owing to these common signs and symptoms, oesophageal achalasia can be misdiagnosed as an eating disorder. In addition, oesophageal achalasia can occur as a complication of BN. It is often difficult to distinguish organic and psychological vomiting or comorbidity because of overlapping of the symptoms.

**Case report:**

We report the case of a patient who developed oesophageal achalasia following severe, repetitive vomiting as a complication of BN.

**Conclusion:**

We want to raise awareness regarding the development of a medical illness in the presence of a psychiatric disorder. Importantly, physicians should have a fundamental knowledge of these two diseases regarding their clinical patterns to differentially diagnose one or both disorders as quickly as possible.

## Introduction

Bulimia nervosa (BN) is an eating disorder manifested by binge eating attacks followed by recurrent inappropriate compensatory behaviours. These behaviours can include self-induced vomiting, excessive exercise or misuse of laxatives, diuretics and enemas in order to prevent weight gain. Long-standing BN may lead to medical complications including oesophageal rupture.^1^ Achalasia is characterised by oesophageal aperistalsis and increased resting pressure with partial or incomplete relaxation of the lower oesophageal sphincter.^2^ Patients have progressive difficulty in swallowing and other symptoms can begin very slowly like chest pain, regurgitation of swallowed food and liquid, heartburn and weight loss. Studies have shown that it is a rare disease with an annual incidence of 1:100 000 individuals and a prevalence of 10:100 000.^3^ Its diagnosis is based on history followed by seriography and oesophageal manometry.^4^ Dysphagia, regurgitation, vomiting, retrosternal pain, heartburn, weight loss, avoidance of eating, consumption of large amount of liquids and aberrant eating behaviours are overlapping symptoms of achalasia and BN.^5^ The overlapping of symptoms can lead to oesophageal achalasia being misdiagnosed as BN.^6^ In addition, achalasia can develop as a complication of BN.

Here we report of the case of a patient who developed achalasia, as a complication of BN, because of chronic and repetitive self-induced vomiting episodes. Our objective is to draw attention that these two disorders may trigger each other or mimic an eating disorder that can lead to misdiagnosis.

## Case report

A 13-year-old adolescent girl was admitted to the Department of Child and Adolescent Psychiatry with complaints of lack of enjoyment of pleasurable activities, sleep disturbances, suicidal thoughts, binge eating episodes, self-induced vomiting episodes and excessive exercising, leading to significant weight loss last 1 year in association with a situation of a family conflict.

The patient lived with her mother and sister. She was very distressed and unhappy because her mother forced her to talk with her father. She stated that her father had deserted them for someone else 9 years ago, she did not talk and see him until this time anymore and she found this difficult to accept him and everyday her father phoned her mother and insisted on establishing a new relationship. In this month, she began to take money from home without permission, especially after each time her father called her mother. And she started to have conflicts with her peers and siblings.

She was admitted to the paediatric inpatient clinics in two different hospitals because of self-induced vomiting episodes and weight loss before admission to the Department of Child and Adolescent Psychiatry. She had no complaints of dysphagia, retrosternal pain and heartburn; physical examinations and detailed investigations including blood tests and abdominal-pelvic ultrasound and endoscopy were normal on both paediatric occasions. Paediatric gastroenterology specialist reported that this medical condition was not associated with any primary oesophageal motility disorders (achalasia, diffuse oesophageal spasm, nutcracker oesophagus, etc.) and secondary oesophageal motility disorders (diabetes mellitus, scleroderma, etc.). Following this, she was referred to a child and adolescent psychiatry outpatient clinic along with a pre-diagnosis of psychiatric disorders to be able to associate with secondary oesophageal motility disorders.

At the psychiatric evaluation, she expressed no fear of getting fat but her self-esteem was strongly affected by her physical appearance, and she seemed very unhappy. She reported binge eating episodes and self-induced vomiting at least 7–8 times a day. She had lost 13 kg in the previous 6 months (weight: 41 kg, height: 162 cm, body mass index: 15.6 at admission). Laboratory evaluations including haemogram, liver function tests, total protein, vitamin B12, folic acid, T3, T4, TSH, FSH, LH, E2, prolactin levels were within normal limits. The abdominal-pelvic ultrasound and plain abdominal radiography were repeated and reported as normal. A physical examination at the paediatric clinic ruled out medical complications. Achalasia and other oesophageal motility disorders were ruled out according to physical examinations and test results. Baseline psychiatric evaluation with the Children’s Depression Inventory and the Clinical Global Impression scale revealed scores of 40 and 7 (extremely ill), respectively. According to those evaluations and DSM-IV-TR criteria, the patient was diagnosed with major depressive disorder and BN, and she was started on fluoxetine 20 mg per day. Cognitive behavioural therapy focusing on body-focused cognitions was also started. Partial response to treatment was observed at the 11th week (i.e. binge or vomiting reduced to 2–3 per day and weight gain of 1.4 kg), and fluoxetine was titrated to 40 mg/day. The subject of seeing her father was closed. She stated that this made her feel good, and she registered for a painting course.

While she was under follow-up in a child and adolescent psychiatry clinic, she was admitted to a paediatric gastroenterology clinic because of complaints of dysphagia with retrosternal pain, heartburn, involuntary vomiting of undigested food and weight loss. Oesophagogastrodu-odenoscopy showed retention of liquid in the oesophagus, so oesophageal manometry was quickly carried out, and the results were strongly consistent with achalasia in the patient. After a joint meeting of the paediatric gastroenterology and paediatric surgery departments, a decision to operate was made. The patient underwent surgery, and oesophagogastromyotomy and fundoplication were performed during the operation. After surgery, her vomiting decreased. And she stated that she felt better but she still experienced anxiety regarding her physical appearance and weight. She was receiving 40 mg/day fluoxetine and cognitive behavioural therapy sessions weekly, and she is still under follow-up. Her Children’s Depression Inventory and the Clinical Global Impression scale scores were 25 and 2 (borderline mentally ill), respectively, during her last visit.

## Discussion

In achalasia, the absent or diminished relaxation of the lower oesophageal sphincter and absent peristalsis of the tubular oesophagus in response to swallowing are thought to be because of the loss of inhibitory nitrinergic neurons in the oesophageal myenteric plexus. The cause of this loss is unclear, and genetic, autoimmune or infectious origins of neuronal damage have been suggested.^2^ As there is not any available cure for achalasia, the treatment approach is mainly focused on allevation of symptoms by pharmacological intervention and surgical approach to lessen the pressure in lower oesophageal sphincter.

BN itself also presents with abnormalities in gastrointestinal physiology.^7,8,9^ Many BN patients present with symptoms such as constipation, nausea, bloating and abdominal pain.^9^ These symptoms are thought to arise from increased gastric capacity and delayed gastric emptying in patients as shown by electrogastrography and radioisotope marker tracing techniques.^10^ Also, postprandial cholecystokinin and stomach relaxation after food consumption are known to be diminished in BN.^11,12^ In patients with BN, symptoms of eating disorder may predispose to achalasia through decreasing gastric motility and triggering myenteric plexus damage because of recurrent vomiting.^13,14^ In addition, previous studies have suggested that these two disorders may trigger each other and create a vicious cycle.^14^

The differentiation between eating disorders and achalasia can be especially difficult because of the complicated crosstalk between the pathophysiology of these disorders. During the follow-up of eating disorders, changes in the symptoms’ frequency, severity and characteristics may be clues to a differential diagnosis and the onset of new complications.^13,14^ In the literature, a study reported that the rate of achalasia was elevated among patients with BN compared to the general population,^7^ and there are some case reports about BN patients developing achalasia because of myenteric plexus damage.^13,14^

In the present case, although response was achieved through psychological support, the sudden onset of the aggravation of symptoms along with involuntary vomiting, heartburning and pain, which was not previously experienced, prompted referral to a paediatric gastroenterology department, and the presence of achalasia was revealed. Therefore, we thought that achalasia may have developed in association with vomiting because of BN. In conclusion, a differential diagnosis of achalasia and eating disorders is not always obvious, and it should be kept in mind that BN may coexist with achalasia, may be misdiagnosed and be complicated by achalasia during follow-ups as in this case.^6,13,14^

Thus, physicians should assess the possibility of oesophageal achalasia in patients presenting with difficulty in swallowing and dysphagia along with symptoms indicating anorexia nervosa or bulimia nervosa.
